# Proteomic Analysis of Responsive Proteins Induced in Japanese Birch Plantlet Treated with Salicylic Acid

**DOI:** 10.3390/proteomes2030323

**Published:** 2014-07-01

**Authors:** Hiromu Suzuki, Yuya Takashima, Futoshi Ishiguri, Nobuo Yoshizawa, Shinso Yokota

**Affiliations:** Department of Forest Science, Faculty of Agriculture, Utsunomiya University, 350 Mine-machi, Utsunomiya, Tochigi 321-8505, Japan; E-Mails: skaku@ba2.so-net.ne.jp (H.S.); ytakashima@ffpri.affrc.go.jp (Y.T.); ishiguri@cc.utsunomiya-u.ac.jp (F.I.)

**Keywords:** Japanese birch, *Inonotus obliquus*, salicylic acid

## Abstract

The present study was performed to unravel the mechanisms of systemic acquired resistance (SAR) establishment and resistance signaling pathways against the canker-rot fungus (*Inonotus obliquus* strain IO-U1) infection in Japanese birch plantlet No.8. Modulation of protein-profile induced by salicylic acid (SA)-administration was analyzed, and SA-responsive proteins were identified. In total, 5 specifically expressed, 3 significantly increased, and 3 significantly decreased protein spots were identified using liquid chromatography/tandem mass spectrometry (LC/MS/MS) and the sequence tag method. These proteins were malate dehydrogenase, succinate dehydrogenase, phosphoglycerate kinase, diaminopimalate decarboxylase, arginase, chorismate mutase, cyclophilin, aminopeptidase, and unknown function proteins. These proteins are considered to be involved in SAR-establishment mechanisms in the Japanese birch plantlet No 8.

## 1. Introduction

Japanese birch (*Betula platyphylla* var. *japonica*) belongs to the Betulaceae family and is distributed throughout the subalpine zone in Honsyu and Hokkaido, Japan. This tree is a pioneer species, and its growth is so fast that the tree is considered to be useful for biomass production [1]. Its sap is used for cosmetics and drinks. In addition, its extractives exhibit potential antioxidant and anti-cancer properties [2]. Its bark contains betulin and betulic acid the derivatives of which possess a wide spectrum of biological and pharmacological activities [3].

*Inonotus obliquus* (Persoon: Fries) Pilat is a white-rot fungus, causes stem heart rot of birch, and produces a black solid sclerotium referred to as sterile conk or canker-like body [4,5]. The endo-polysaccharide extracted from the mycelia of the fungus exhibits indirect anti-cancer effects [6].

Infected plant tissues induce various resistance responses, formation of physical barriers (papilla, thick cell wall, lignification, *etc.*), accumulation of antipathogenic compounds (phenolic compounds, phytoalexins, pathogenesis-related (PR) proteins, reactive oxygen species (ROS), *etc.*), and hypersensitive cell death named hypersensitive reaction (HR) [7]. The papilla is the reinforced cell wall apposition localized at the site of fungal penetration, where phenolic compounds and callose are accumulated [7]. Lignin gives the cell wall physical strength and has an antipathogenic activity [8]. Phytoalexins are low molecular weight compounds accumulated in plant tissues by pathogen infection that have an antipathogenic activity [9]. More than 200 compounds are identified as phytoalexins in various plant species [7]. PR proteins are newly expressed upon infection, although not necessarily under all pathological conditions [10]. PR proteins are divided into several groups based on their sequence and potential functions [10]. Their biochemical properties include glucanase, chitinase, peroxidase, and protease activities. Some of them have unknown functions and indirect antipathogenic activities [11]. HR is considered as one of the phenomena for programmed cell death (PCD) induced by pathogen infection. It inhibits obligate parasite growth and diffusion. Growth of other pathogens is also inhibited by antipathogenic compounds accumulated during HR induction [7]. ROS play important roles in defense responses. They act as antipathogenic and signaling compounds in plants [12]. The above-mentioned physical barriers and antipathogenic compounds inhibit pathogen growth and, consequently, induce pathogen resistance.

It is known that pathogen resistance is not only induced in infected tissues but also in systemic tissues. This resistance, referred to as systemic acquired resistance (SAR), is considered to be important in pathogen resistance reactions. Production of PR proteins is usually observed before an infectious challenge. In contrast, other reactions have been detected after an infectious challenge. For example, accumulation of phenolic compounds and higher activities of peroxidase and chitinase induced by infection of *Colletotrichum lageizarium* were observed with pretreatment of SAR-inducing molecules in *Cucumis sativus* L [13]. Thus, the systemic signals can prepare the systemic tissues for a faster defense response [14]. Salicylic acid (SA) is one of the SAR-inducing molecules that are required for SAR establishment [15]. It has been reported that various other proteins and genes are also expressed by pathogen infection and SA administration [16,17,18]. These proteins are considered to be important for SAR establishment.

Signal transduction mechanisms for establishment of SAR have been investigated mainly by using *Arabidopsis thaliana* and *Nicotiana tabacum* [15,19,20,21,22,23]. According to these studies, it has been proposed that SA induces expression of SAR-associated genes by the following mechanisms. After pathogen recognition by receptors of the plants, rapid and transient ROS production, called oxidative burst, occurs in the tissues. In a next step, the ROS induce an increase in the amount of SA in both the infected and systemic tissues, and the SA changes the cellular redox state and induces thioredoxin expression. The thioredoxin reduces the disulfide bonds in the cellular nonexpresser of PR genes 1 (NPR1) oligomers, one of the SA signal transduction factors. Reduced NPR1 oligomers release NPR1 monomers, that they bind to TGA transcription factors in the nuclei. The TGA transcription factor expresses SA-induced genes. In addition, NPR1-independent pathways also exist [23]. The translocating signal molecules between infected and systemic tissues are essential for SAR, though they have not been determined yet. It has been reported that methyl salicylate (MeSA) is considered to be a possible SAR long-distance signaling compound in *N. tabacum* [24]. However, there is a report demonstrating that SA is not the translocating signal [25] and that MeSA production is not essential for SAR in *A. thaliana* [26]. In addition, it is suggested that peptide and lipid derivatives are possible long-distance signaling molecules [27]. Moreover, a recent study has demonstrated that azelaic acid (AZA) is a possible translocating signal molecule [28].

In our previous studies of pathogen resistance in Japanese birch plantlet No.8, heat shock 60 kDa and 70 kDa proteins were found to be specifically expressed during the infection of *I. obliquus* strain IO-U1 [29]. In addition, in anatomical and histochemical observations, lignification, phenolics deposition, and necrophylactic periderm formation have occurred as infection-induced responses in Japanese birch plantlet No.8 [30] and Tohoku [31] infected with *I. obliquus* strain IO-U1.

Signal transduction mechanisms for the establishment of pathogen resistance including SAR have been investigated using herbaceous plants such as *A. thaliana* and *N. tabacum*, while there are only a few studies on woody plants.

The purpose of the present study was to unravel the mechanisms of SAR establishment and resistance signal transduction pathways against the infection of *I. obliquus* strain IO-U1 in Japanese birch plantlet No.8. The protein profile changes induced by SA-administration were analyzed, and SA-responsive proteins were identified to clarify SAR establishment mechanisms.

## 2. Experimental

### 2.1. Plant Material

Japanese birch (*Betula* *platyphylla* var. *japonica*), obtained from the Forestry and Forest Products Research Institute, Ibaraki, Japan was used as plant material for this study. The plantlets were grown *in vitro* on Murashige and Skoog medium [32] containing 20 g/L sucrose, 2.5 μM indole-3-acetic acid, 0.1 μM 1-naphthalenacetic acid at 25 °C under illumination at 50 μmol∙m^−2^∙s^−1^ for a 16-h photo period during 3 months. Axillary buds were subcultured for propagation of the plantlets on the same medium every 3 months.

### 2.2. Salicylic Acid (SA) Treatment

High purity grade SA (Kanto Chemical Co., Tokyo, Japan) was used for the treatment. SA aqueous solution (0.5 mM) was prepared and its pH was adjusted to around 7.0 with 0.1 M and 0.01 M NaOH. The solution was sterilized with a membrane filter (Millex-GV, 0.22 μM, Millipore, Billerica, MA, USA) before administering it to the plantlets.

In this study, intact (C1), wounded (C2_SA_), and SA-infiltrated (T_SA_) plantlets were prepared. The surface of the third node from the apex of a plantlet was cut into a V-shape with a surgical knife, and then 1 μL ultra-pure water or 1 μL SA aqueous solution was administered to C2_SA_ or T_SA_ plantlets, respectively. After the treatments, the plantlets were further grown for 2 days under the conditions described above.

### 2.3. Preparation of Protein Samples

Protein extraction was repeated three times for each treatment. After 48 h of the treatments, each plantlet was deep-frozen with liquid nitrogen and powdered with a mortar and pestle. In a next step, the extraction buffer was added to the powdered samples in a volume of 1 mL/g fresh weight of the plantlet, and the powdered plantlet was further mashed. The extraction buffer was prepared by mixing the following EXT-1, 2, and 3 in a volume ratio of 3:2:1. EXT-1 was prepared by dissolving the Trizma Preset pH crystal 7.5 (Sigma, St. Louis, MO, USA, 7.54 g), dihydrate disodium salt of ethylenediamine-*N*,*N*,*N’*,*N’*-tetraacetic acid (Doujin, Tokyo, Japan, 0.56 g), and glycerol (100 mL) in ultra-pure water, followed by adjusting to 250 mL. EXT-2 was prepared by dissolving Triton-X-100 (Acros Organics, Geel, Belgium, 6.4 g) in ultra-pure water and heating it at 60 °C, followed by adjusting it to 100 mL. EXT-3 was prepared by dissolving dithiothreitol (DTT, Wako Pure Chemical Co., Osaka, Japan, 463 mg) in ultra-pure water, followed by adjusting to 50 mL. The homogenates were sonicated with a supersonic homogenizer (Sonic Fire 250, Branson, Danbury, CT, USA). The homogenate was centrifuged at 10,000× *g* for 30 min at 4 °C, and the supernatant was collected in another centrifuge tube (SuperClear Centrifuge tube, Labcon, Petaluma, CA, USA). The supernatant was centrifuged again under the same condition, and then the obtained supernatant was collected in a Teflon-lined centrifuge tube (Nalgene, Rochester, NY, USA). Proteins in the supernatants were precipitated with 10% (w/v) trichloroacetic acid (TCA) aqueous solution, and then the solution was incubated at −20 °C for 1 h. After thawing the sample, it was centrifuged at 10,000× *g* for 30 min at 4 °C, and the supernatant was removed. Cold acetone was added to the obtained pellets, and the pellet was mashed and stirred with a spatula. The suspension was centrifuged at 10,000× *g* for 30 min at 4 °C, and the supernatant was removed. This process was repeated three times. The obtained pellets were dried under nitrogen gas. Solubilization buffer (100 μL) was added to the dried pellets, and they were incubated for 30 min. The solubilization buffer was prepared by dissolving urea (GE healthcare, Little Chalfont, Buckinghamshire, UK; 4.204 g), thiourea (GE Healthcare, 1.422 g), 3-[(3-cholamidopropyl)-dimethylammonio]-1-propane sulfonate (CHAPS, GE Healthcare, 400 mg), DTT (30.8 mg), and immobilized pH gradient buffer pH 4–7 (GE Healthcare, 200 μL) in ultra-pure water, followed by adjusting to 10 mL. The suspension was centrifuged at 10,000× *g* for 30 min at 4 °C, and the supernatant was collected in a 1.5 mL micro tube (Eppendorf, hamburg, Germany). This process was repeated two times. The protein content of the obtained sample was determined using the Bradford [33] method using ovalbumin (Sigma) as a standard. The obtained samples were stored at −20 °C before use.

### 2.4. Two-Dimensional Electrophoresis (2-DE)

Two-dimensional electrophoresis (2-DE) was repeated three times for each treatment. Isoelectric focusing (IEF) was carried out as follows. The protein sample (1 mg) was put in a 1.5 mL micro tube, and the total volume was adjusted to 250 μL with rehydration buffer. The rehydration buffer was prepared by dissolving urea (12 g), CHAPS (0.5 g), immobilized pH gradient buffer pH 4–7 (125 μL), and bromophenol blue (BPB, Kanto Chemical Co. Tokyo, Japan) stock solution (50 μL) in ultra-pure water, followed by adjusting to 25 mL. BPB stock solution was prepared by dissolving BPB (100 mg) and tris-base (MP Biomedicals, Irvine, CA USA, 60 mg) in ultra-pure water, followed by adjusting to 10 mL. An Immobiline DryStrip (pH 4–7, 13 cm, GE Healthcare) was rehydrated with the above mixture at 22 °C for at least 10 h on the Ettan IPGphor II (GE Healthcare). In the preliminary experiments, the DryStrip in the pH range of 3–10 was used, and most of the protein spots were recognized in the acidic to neutral range. Thus, the DryStrip in the pH range of 4–7 was used in this study. To prevent the DryStrip from drying, it was covered with 800 μL of Immobiline DryStrip Cover Fluid (GE Healthcare). After rehydration, IEF electrode stripes (5 mm in length, GE Healthcare) were placed between DryStrip and electrodes of a strip holder. IEF was carried out at 20 °C. Maximum current was set at 50 μA per stripe. Voltage program was as follows: step-n-hold, 500 V, 1 h; gradient, 1000 V, 1 h; gradient, 8000 V, 5 h; step-n-hold, 8000 V, 8 h.

After IEF, the DryStrip was treated with 10 mL of SDS-equilibrium buffer containing 1% (w/v) DTT in a 60 mL test tube with a screw cap (IWAKI, Tokyo, Japan) for 30 min. Then the DryStripe was treated with 10 mL of SDS-equilibrium buffer containing 2.5% (w/v) iodoacetoamide in another 60 mL test tube with a screw cap for 30 min in the dark. The DryStripe was shaken in 10 mL of SDS-running buffer in another 60 mL test tube with a screw cap to remove remaining equilibrium buffer. The DryStripe was put on filter paper infiltrated with ultra-pure water to remove remaining SDS-running buffer.

SDS-polyacrylamide gel electrophoresis (SDS-PAGE) was carried out using SE600 Ruby (GE healthcare) as follows. Polyacrylamide gel was prepared at 12.5% acrylamide concentration and 1.5 mm in thickness. Running buffer was cooled with thermostatic circulator (Multi temp III, GE healthcare) set at 10 °C. The equilibrated DryStripe was fixed at the top of gel with 1 mL of agarose gel. A marker bead was prepared by mixing 60 μL of agarose gel with 30 μL of molecular weight marker (Precision Plus Protein Standards, Bio-Rad, Hercules, CA, USA). The marker bead was placed on the side end of DryStripe. Electrophoresis was carried out at 600 V of maximum voltage and 15 mA of constant current per gel for 15 min. Then constant current was set at 30 mA per gel, and electrophoresis was carried out until BPB reached the bottom of the gel.

### 2.5. Staining 2-DE Gel

The 2-DE gel was stained with Coomassie Brilliant Blue (CBB) as follows. The gel was shaken in 250 mL of CBB (PhastGel Blue R-350, GE Healthcare) staining solution for 3 h. After that the 2-DE gel was shaken in 250 mL of destaining solution two times for 10 and 3 h, respectively. The destaining solution was prepared by mixing 150 mL ethanol and 50 mL acetic acid with 300 mL distilled water. In the case of image analysis of 2-DE gel, the destained gel was shaken in 250 mL of preservation solution for 30 min twice. The preservation solution was prepared by dissolving 150 mL ethanol and 23 mL glycerol in distilled water, followed by adjusting to 500 mL with distilled water. The 2-DE gel was preserved with Gel Drying kit (Promega, Madison, WI, USA).

### 2.6. Image Analysis of 2-DE Gels

The 2-DE gel images were captured with a scanner (GT-9700, EPSON, Gen, Suwa, Japan) at a 300 dpi resolution and analyzed with ImageMaster 2D Platinum ver. 5.0 (GE Healthcare). Protein spots without reproduction were excluded from the analysis. Protein spots only expressed in each treatment were considered as specifically expressed ones. Those protein spots were analyzed, which were considered as specifically expressed in T_SA_ gel and were expressed in T_SA_ gel in significantly different amounts compared to the corresponding spots in both C1 and C2_SA_ gels. These protein spots were regarded as SA-responsive protein spots. Protein expression was evaluated in percent intensity for statistical significance (*p* < 0.05) using Student’s *t*-test provided with the software.

### 2.7. In-Gel Digestion

In-gel digestion of the protein spots was carried out as follows: The spots of SA-responsive proteins were cut out from stained gels of T_SA_ and put in 1.5 mL micro tubes (Eppendorf, Hamburg, Germany). The cut gel was treated with 200 μL of destaining solution under agitation for 10 min to overnight. The gel was soaked in 100 μL of acetonitrile under agitation for 5 min. The acetonitrile was removed, and the gel was dried with a centrifugal evaporator (CE-1, Hitachi, Tokyo, Japan) for 15 min. The gel was infiltrated in 100 μL of reducing solution (10 μL of 1 M DTT solution and 25 μL of 1 M ammonium hydrogen carbonate in 965 μL ultra-pure water) under agitation at 56 °C for 1 h. The gel was washed with 100 μL of washing buffer (75 μL of 1 M ammonium hydrogen carbonate in 2925 μL ultra-pure water) under agitation for 10 min. The gel was treated with 100 μL of alkylating solution (10 mg iodeacetamide (Wako Pure Chemical Co., Osaka, Japan) in 1 mL washing buffer) under agitation for 45 min in the dark. The gel was washed with 100 μL of washing buffer under agitation for 10 min. The gel was washed with 200 μL of washing solution (5 mL methanol and 5 mL of 10% acetic acid) under agitation four times at least for 1 h. The gel was soaked in 100 μL of equilibration buffer (100 μL of 1 M ammonium hydrogen carbonate in 900 μL ultra-pure water) under agitation for 5 min. The gel was treated with 200 μL of dehydration solution (2 mL acetonitrile and 100 μL of 1 M ammonium hydrogen carbonate in 1.9 mL ultra-pure water) under agitation twice for 10 min. The dehydration solution was removed, and the gel was dried using a centrifugal evaporator for 15 min. The gel was incubated in 20 μL of trypsin (gold mass spectrometry grade, Promega) solution on ice for 30 min. The excessive trypsin solution was removed, and the gel was incubated at 37 °C overnight. The gel was sonicated in 50 μL of extraction buffer (mixture of 500 μL acetonitrile and 500 μL 0.1% trifluroacetic acid (Wako Pure Chemical Co.)) using an ultrasonicator (3510, Branson) for 10 min. The extract was collected in a 1.5 mL micro tube. This process was repeated again with 25 μL of extraction buffer. The collected peptide sample was dried with a centrifugal evaporator until it was completely dried. The peptide sample was desalted with ZipTip μ-C18 (Millipore, Billerica, MA, USA).

### 2.8. LC/MS/MS of the Peptide Sample

A nanospray LTQ XL Orbitrap MS (Thermo Fisher Scientific, Waltham, MA, USA) was operated in data-dependent acquisition mode using the installed XCalibur software. Using an Ultimate 3000 nanoLC (Dionex, Sunnyvale, CA, USA), peptide samples in 0.1% formic acid were loaded onto a 300 μm ID × 5 mm C18 PepMap trap column. The peptide samples were eluted from the trap column and their separation and spraying were done using 0.1% formic acid in acetonitrile at a flow rate of 200 nL/min on a nano-capillary column (NTTC-360, Nikkyo Technos, Tokyo, Japan) with a spray voltage of 1.8 kV. Full scan mass spectra were obtained in the orbitrap over 150–2000 *m*/*z* with a resolution of 15,000. The three most intense ions above the 1000 threshold were selected for collision-induced fragmentation in the linear ion trap at a normalized collision energy of 35% after accumulation to a target value of 1000. Dynamic exclusion was employed within 30 s to prevent repetitive selection of peptides. Obtained MS/MS spectra were converted to individual DTA files using BioWorks software ver. 3.3.1 (Thermo Fisher Scientific). The following parameters were set to create a list of peaks: parent ions in the mass range with no limitation, one grouping of MS/MS scans, and the threshold at 100.

### 2.9. Database Search

The database search was carried out by sequence tag method with the MASCOT search engine [34] (Matrix Science, Boston, MA, USA) using obtained data files. The proteins with the highest scores were considered. The following parameters were set for the search: Database, NCBInr; taxonomy, green plants; digest enzyme, trypsin; maximum missed cleavage, one; fixed modification, carbamidomethylation of cysteine; variable modification, oxidation of methionine; peptide mass tolerance, 10 ppm; fragment mass tolerance, 0.2 Da; peptide charge, +1, +2, +3; statistical significance, *p* < 0.05. 

## 3. Results and Discussion

3.1. 2-DE and Image Analysis

**Figure 1:**
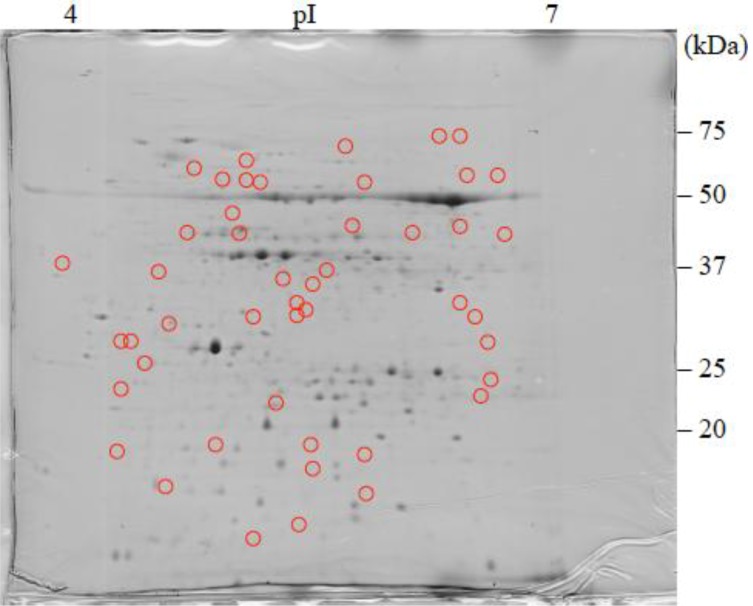
Coomassie Brilliant Blue (CBB) stained two-dimensional electrophoresis gel of C1 plantlet. 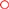: Specifically expressed protein spots in C1 gel.

**Figure 2:**
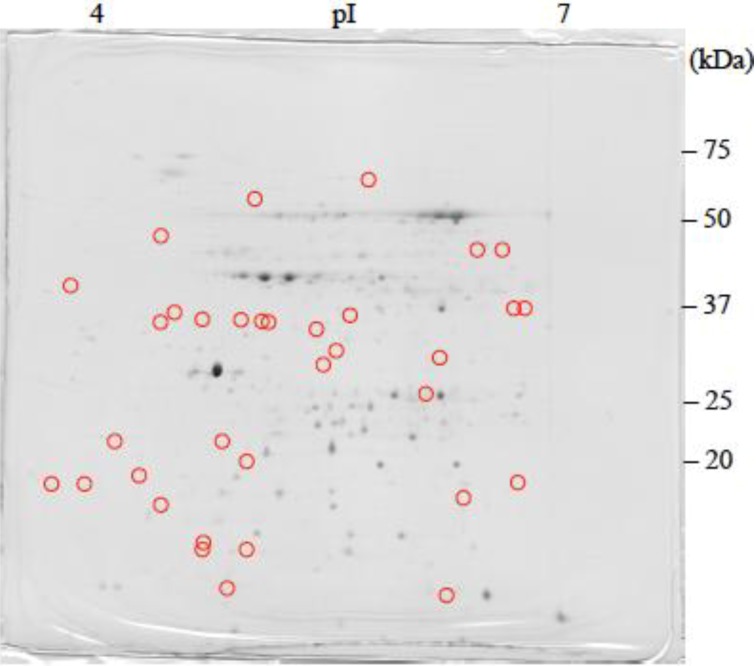
CBB stained two-dimensional electrophoresis gel of C2_SA_ plantlet. 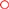: Specifically expressed protein spots in C2_SA_ gel.

**Figure 3:**
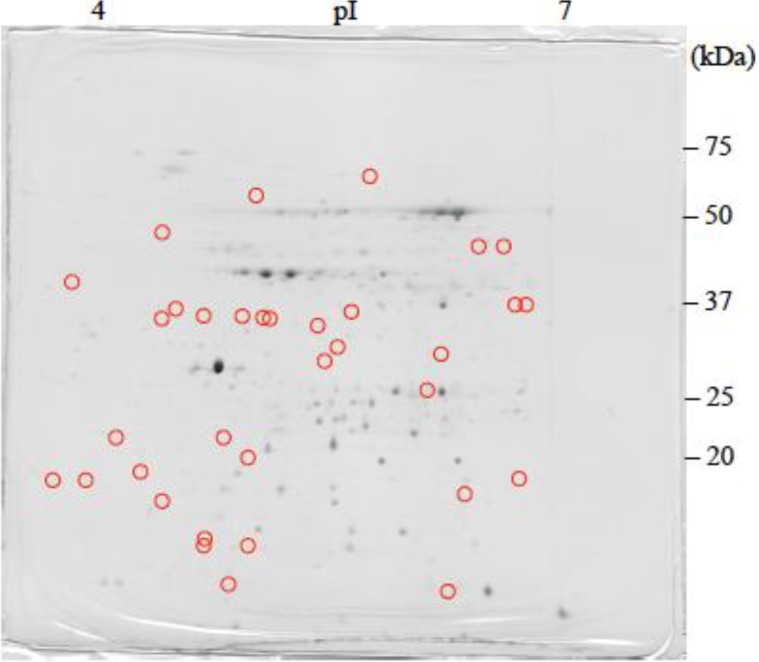
CBB stained two-dimensional electrophoresis gel of T_SA_ plantlet. 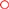: Specifically expressed protein spot in T_SA_ gel. 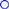: Significantly increased protein spots in T_SA_ gel. 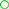: Significantly decreased protein spots in T_SA_ gel.

**Figure 4:**
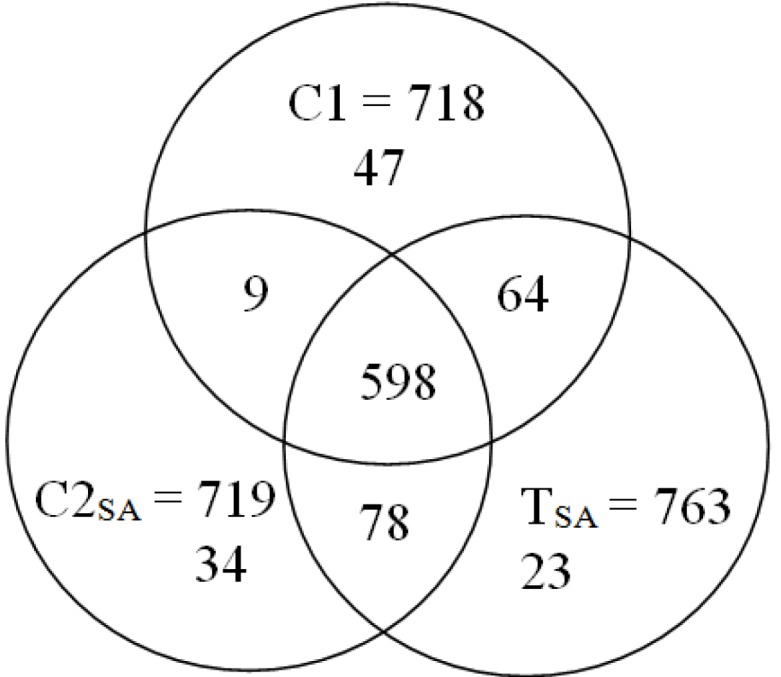
The number of reproducing spots detected in each treatment.

**Figure 5:**
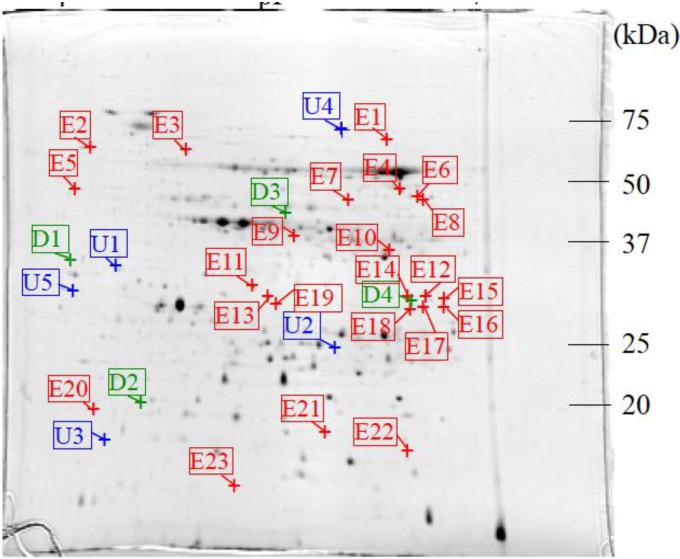
IDs of salicylic acid (SA)-responsive protein spots. 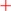: Specifically expressed protein spots in T_SA_ gel; 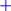: Significantly increased protein spots in T_SA_ gel; 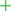: Significantly decreased protein spots in T_SA_ gel.

The 2-DE gel images are shown in Figure 1, Figure 2 and Figure 3. Of these spots, the numbers of total reproducing spots in C1, C2_SA_, and T_SA_ gels were 718, 719, and 763, respectively (Figure 4). The numbers of specifically expressed spots were 47, 34, and 23, respectively. IDs were designated to each SA-responsive protein spot (Figure 5).

Most of the T_SA_-specifically-expressed spots were detected in the ranges of pI 5.5–7.0 and 30–50 kDa on the gel (Figure 3). C1-specifically-expressed spots were more frequently detected on the acidic side (Figure 1). C2_SA_-specifically-expressed spots were detected in the middle ranges of the gel (Figure 2).

**Figure 6:**
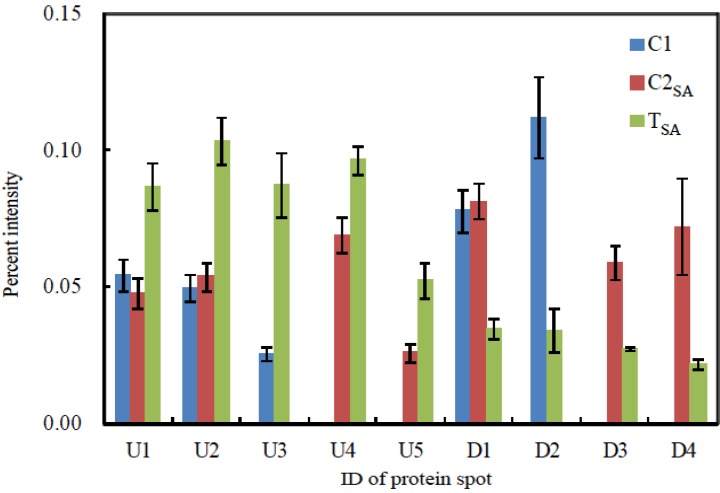
Mean spot percent intensities of significantly changed proteins. Bar represents mean ± standard error of percent intensity of each protein spot in three repetitive gels from different plantlets.

Figure 6 shows the mean spot percent intensities of significantly increased or decreased proteins. The numbers of significantly increased spots in the T_SA_ gel compared to both C1 and C2_SA_ gels, to the C1 gel, and to the C2_SA_ gel were two, one, and two, respectively. The numbers of significantly decreased spots in the T_SA_ gel compared to both the C1 and C2_SA_ gels, to the C1 gel, and to the C2_SA_ gel were one, one, and two, respectively. The U1 spot of T_SA_ increased about two-fold compared to both C1 and C2_SA_. The U3 and U5 of T_SA_ increased more than two-fold compared to C1 and C2_SA_, respectively. The D1 decreased to less than one half compared to both C1 and C2_SA_. The D2, D3, and D4 decreased to less than one half compared to C1 or C2_SA_.

### 3.2. Protein Identification

**Figure 7:**
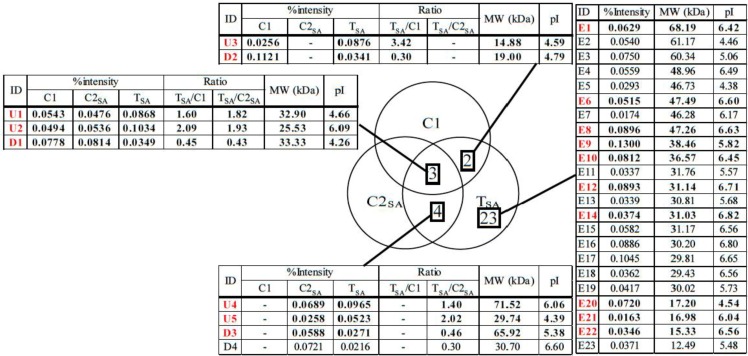
The number of SA responsive protein spots. The IDs in red color correspond to the protein spots analyzed by liquid chromatography/tandem mass spectrometry (LC/MS/MS). Percent intensity: The mean percent intensity of each SA-responsive protein spot in T_SA_ gel. T_SA_/C1, T_SA_/C2_SA_: The ratios of the mean percent intensity of each SA-responsive protein spot in T_SA_ gel to the mean percent intensity of each corresponding protein spot in C1 and C2_SA_ gels.

In total, 5 specifically expressed, 3 significantly increased, and 3 significantly decreased spots were identified by liquid chromatography/tandem mass spectrometry (LC/MS/MS) (Figure 7) and the sequence tag method (Table 1). These proteins were categorized into four groups: energy production, metabolism, protein synthesis, and unknown function.

**Table 1:** Identified proteins by LC/MS/MS and sequence tag method.

ID	Protein	No. in NCBI	Score	% cover	Mw (kDa) Observed/Theoretical	pI Observed/Theoretical	Ratio		Plant
							T_SA_/C1	T_SA_/C2_SA_	
Energy production									
E10	Malate dehydrogenase	gi|255585544	139	14	36.57/28.84	6.45/5.44	-	-	*Ricinus communis*
U4	SDH1-1; ATP binding/succinate dehydrogenase	gi|15240075	43	2	71.52/70.24	6.06/5.86	-	1.40	*Arabidopsis thaliana*
D3	Phosphoglycerate kinase, chloroplastic; Flags; Precursor	gi|2499497	85	11	65.92/50.31	5.38/8.48	-	0.46	*Nicotiana tabacum*
Metabolism									
E6	Diaminopimelate decarboxylase putative	gi|255543757	79	3	47.34/54.55	6.62/6.58	-	-	*Ricinus communis*
E9	Arginase	gi|148828535	440	21	42.29/37.15	5.76/6.14	-	-	*Malus hupehensis*
U1	Chorismate mutase precursor	gi|429153	39	2	32.90/38.05	4.66/5.90	1.60	1.82	*Arabidopsis* *thaliana*
Protein synthesis									
E22	Peptidylprolyl isomerase (cyclophilin)	gi|21886603	44	8	15.33/18.51	6.56/8.68	-	-	*Betula pendula*
U5	Aminopeptidase	gi|255080640	35	1	29.74/63.62	4.39/7.97	-	2.02	*Micromonas* sp. RCC299
Unknown									
E12	Predicted protein	gi|168008150	23	1	31.14/71.46	6.71/8.15	-	-	*Physcomitrella patens* subsp. *Patens*
D1	Hypothetical protein	gi|2342730	16	1	33.33/54.76	4.26/6.28	0.45	0.43	*Arabidopsis thaliana*
D2	Predicted: hypothetical protein	gi|225432620	101	14	19.00/22.52	4.79/9.73	0.30	-	*Vitis vinifera*

Note: T_SA_/C1, T_SA_/C2_SA_: The ratios of the mean percent intensity of each SA-responsive protein spot in T_SA_ gel to the mean percent intensity of each corresponding protein spot in C1 and C2_SA_ gels.

#### 3.2.1. The Proteins Related to Energy Production

The E10 protein was identified as malate dehydrogenase (MDH). MDH catalyzes the oxidation of malate to oxaloacetate, producing NADH [35]. This protein was induced in *C. sativus* L. cv Negrito by *Trichoderma asperellum* inoculation with increase in SA [36]. *Phellinus sulphurascens* inoculation also induced this protein in *Pseudotsuga menziesii* [37]. Mitochondrial MDH lowers leaf respiration and alters photorespiration and plant growth by partitioning carbon and energy in leaves of Arabidopsis [38]. In addition, it was reported that over-expression of cytosolic NAD-dependent MDH gene resulted in promotion of growth and tolerance to cold and high salinity in apple callus and tomato [39]. Thus, MDH expression may increase the resistance by altering the distribution of carbon and energy in Japanese birch plantlet No.8 treated with SA.

The U4 protein was identified as SDH1-1; ATP binding/succinate dehydrogenase. Succinate dehydrogenase catalyzes the oxidation of succinate to fumarate by reducing FAD to FADH_2_ in the citric acid cycle [40]. This protein expression was induced in a *Hordeum vulgare* L.-susceptible line by *Erysiphe graminis* inoculation [41], and some isozymes were expressed in *A. thaliana* seeds with SA administration [16]. FADH_2_ has an important role in oxidation-reduction reactions as well as NADPH metabolism. Increase of this protein may also increase FADH_2_ production and supply fumaric acid to the TCA cycle leading to the production of resistant compounds in Japanese birch plantlet No.8.

D3 was identified as phosphoglycerate kinase. Phosphoglycerate kinase participates in the glycolytic pathway. This enzyme catalyzes the formation of 3-phosphoglycerate from 1,3-bisphosphoglycerate [42]. Some isozyme expressions were decreased in *A. thaliana* seeds and *G. barbadense* seedlings with SA administration and *Verticillium dahliae* infection, respectively [16,18]. In the case of *G. barbadense*, phosphoglycerate kinase expression decreased, whereas the expressions of other enzymes related to the glycolytic pathways increased. It is, therefore, proposed that the expression change of these proteins redirects the metabolic flux from the glycolysis to the pentose phosphate pathway, producing more intermediates, such as 1-deoxy-D-xylulose 5-phosphate for the generation of isoprenoid [19]. Isoprenoids are known to be important antipathogenic compounds. Decrease of this enzyme, therefore, may affect the biosynthetic pathways of antipathogenic compounds in Japanese birch plantlet No.8.

#### 3.2.2. The Proteins Related to Metabolism

The E6 protein was identified as diaminopimelate decarboxylase. This enzyme decarboxylates *meso*-2,6-diaminopimelic acid to lysine in the diaminopimelic acid pathway [43]. The function of this enzyme on pathogen resistance is not known. Increase of this protein may increase lysine production, and lysine may be used for synthesis of other proteins involved in producing antipathogenic and/or signaling compounds in Japanese birch plantlet No.8.

The E9 protein was identified as arginase. This enzyme hydrolyzes L-arginine to L-ornithine and urea [44]. This activity and the expression of this enzyme are increased by wounds, treatment with jasmonic acid or coronatine [45], and *P. syringae* infection [46]. It is suggested that this protein has an important role for polyamine production, because L-ornithine is a precursor for polyamine. Polyamines are aliphatic hydrocarbons possessing more than two amino groups, and they have various physiological activities [47]. Polyamines also play an important role in plant-pathogen interactions. Polyamines are usually conjugated with cinnamic acids, and the resulting conjugates are known as hydroxycinnamic acid amides (HCAAs). It has been reported that good correlations exist between the accumulation of HCCAs and pathogen resistance [48]. Modulation of host polyamine levels has led to significant changes in host susceptibility to different kinds of pathogens in *N. tabacum* [49]. In addition, polyamine is a source of H_2_O_2_ through its degradation, and it induces hypersensitive response-like cell death in *N. tabacum* [50]. It has been reported that SA treatment increases the amount of putrescine, one of polyamines in *Zea mays* L., hybrid Norma [51]. This protein expression induced by SA may lead to an increase polyamine production through an increase of L-ornithine in Japanese birch plantlet No.8.

The U1 protein was identified as chorismate mutase. This enzyme transforms chorismic acid to prephenic acid in the shikimic acid pathway [52]. This enzyme expression was increased by elicitor treatment and infections with *Fusarium oxysporum* and *Alternaria raphani* in *A. thaliana* [53]. Phenylalanine derived from the shikimic acid pathway is a precursor of important compounds for defense responses, such as phenolic compounds containing monolignols and isoflavonoids [54]. SA is also derived from phenylalanine, and it has been suggested that the SA-mediated signaling pathway is regulated by a positive feedback loop [55,56,57]. In addition, SA treatment has increased the amount of phenolic compounds and induced SAR in *Vigna mungo* [20]. Increase of this protein expression is considered to increase the amount of phenolic compounds including SA for antipathogenic responses and signaling pathways in Japanese birch plantlet No.8.

#### 3.2.3. The Proteins Related to Protein Synthesis

The E22 protein was identified as cyclophilin. This protein catalyzes rotation of proline-peptide bonds and is considered to participate in the protein folding process as a chaperone [58,59]. This protein expression was induced by various biotic and abiotic stresses in *Phaseolus vulgaris* L. cv. Saxa. As SA administration also induces its expression, it has been proposed that cyclophilin may function as a chaperone-like molecule in diseased plants in order to decrease the risks of degradation or to avoid aggregation of proteins, the reactions that take place under stress [60]. In this study, cyclophilin was induced, suggesting that this protein folds pathogen-induced proteins and protects other proteins from degradation and aggregation in Japanese birch plantlet No.8.

The U5 protein was identified as aminopeptidase. This protein family removes an amino acid from the N-terminal of a peptide, and includes leucine aminopeptidase, prolineiminopeptidase, serine aminopeptidase, and so on [61]. The aminopeptidase family protein and leucin aminopeptidase were increased in their expression with SA treatment in *A. thaliana* suspension culture [62]. Expression of aminopeptidase was also increased with *P. syringae* DC3000 inoculation in *A. thaliana* [46]. SA involves in the interactions between *A. thaliana* and *P. syringae* DC3000 [63]. N-terminal residues of the protein are correlated with a protein half-life through degradation by S26 proteasome in animals, yeast, and prokaryotes. In addition, it has been suggested that this mechanism is also active and important in plants. The ability of aminopeptidases to remove N-terminal residues and to reveal penultimate residues could influence a protein’s interactions between the N-terminal residues and the ubiquitin-proteasomal system, and could, therefore, influence protein stability [61]. In addition, it has been reported that cysteine proteases play an instrumental role in programmed cell death (PCD) triggered by oxidative stress in *Glycine max* [64]. As oxidative stress is correlated with the SA signaling pathway [21], it is suggested that SA is involved in the degradation of proteins induced by PCD. The increased aminopepetidase found in this study may influence protein stability and signaling pathways by removing the N-terminal residues of proteins in Japanese birch plantlet No.8.

#### 3.2.4. Unknown Function Protein

The E12 was identified as predicted protein. In addition, the D1 and D2 were identified as hypothetical proteins. Although their functions are unknown, changes in expression of these proteins may be involved in SAR establishment in Japanese birch plantlet No.8.

## 4. Conclusions

In this study, protein profile changes induced by SA-treatment were analyzed, and SA-responsive proteins were identified to unravel the mechanisms of SAR establishment in Japanese birch plantlet No.8. In total 5 specifically expressed, 3 significantly increased, and 3 significantly decreased proteins were identified by LC/MS/MS and sequence tag method. These proteins were categorized into energy production, metabolism, protein synthesis, and unknown function, and were considered to be involved in SAR establishment in Japanese birch plantlet No.8.

## References

[1] Hosoi Y., Ohyama K., Saito A. (1989). *Betula platyphyll*a var. *japonica*. Current Biotechnology Complete Book: Propagation and Breeding of Woody Plant.

[2] Ju E.M., Lee S.E., Hwang H.J., Kim J.H. (2004). Antioxidant and anticancer activity of extract from *Betula platyphylla* var. *japonica*. Life Sci..

[3] Sami A., Taru M., Salme K., Jari Y.K. (2006). Pharmacological properties of the ubiquitous natural product betulin. Eur. J. Pharm. Sci..

[4] Zabel R.A. (1976). Basidiocarp development in *Inonotus obliquus* and its inhibition by stem treatment. Forest Sci..

[5] True R.P., King J.F. (1995). Cankers and decays of birch associated with two *Poria* species. J. Forest.

[6] Kim Y.O., Park H.W., Kim J.H., Lee J.Y., Moon S.H., Shin C.S. (2006). Anti-cancer effect and structural characterization of endo-polysaccharide from cultivated mycelia of *Inonotus obliquus*. Life Sci..

[7] Schumann G.L., D’Arcy C.J. (2006). Induced (active) defenses. Essential Plant Pathology.

[8] Boerjan W., Ralph J., Baucher M. (2003). Lignin biosynthesis. Annu. Rev. Plant Biol..

[9] Lattanzio V., Lattanzio V.M.T., Cardinali A., Maeda N. (2006). Role of phenolics in the resistance mechanisms of plants against fungal pathogens and insects. Neural Proteoglycan.

[10] Van Loon L.C., Rep M., Pieterse C.M.J. (2006). Significance of inducible defense-related proteins in infected plants. Annu. Rev. Phytopathol..

[11] Ferreira R.B., Monteiro S., Freitas R., Santos C.N., Chen Z., Batista L.M., Duarte J., Borges A., Teixeira A.R. (2007). The role of plant defence proteins in fungal pathogenesis. Mol. Plant Pathol..

[12] Mehdy M.C. (1994). Active oxygen species in plant defense against pathogens. Plant Physiol..

[13] Siegrist J., Jeblick W., Kauss H. (1994). Defense responses in infected and elicited cucumber (*Cucumis sativus* L.) hypocotyl segments exhibiting acquired resistance. Plant Physiol..

[14] Métraux J.P., Nawrath C., Genoud T. (2002). Systemic acquired resistance. Euphytica.

[15] Gaffney T., Friedrich L., Vernooij B., Negrotto D., Nye G., Uknes S., Ward E., Kessmann H., Ryals J. (1993). Requirement of salicylic acid for the induction of systemic acquired resistance. Science.

[16] Rajjou L., Belghazi M., Huguet R., Robin C., Moreau A., Job C., Job D. (2006). Proteomic investigation of the effect of salicylic acid on *Arabidopsis* seed germination and establishment of early defense mechanisms. Plant Physiol..

[17] Kundu S., Chakraborty D., Pal A. (2011). Proteomic analysis of salicylic acid induced resistance to mungbean yellow mosaic India virus in *Vigna mungo*. J. Proteomics.

[18] Wang F.X., Ma Y.P., Yang C.L., Zhao P.M., Yao Y., Jian G.L., Luo Y.M., Xia G.X. (2011). Proteomic analysis of the sea-island cotton roots infected by wilt pathogen *Verticillium dahliae*. Proteomics.

[19] Cao H., Bowling S.A., Gordon A.S., Dong X. (1994). Characterization of an *Arabidopsis* mutant that is nonresponsive to inducers of systemic acquired resistance. Plant Cell.

[20] Alverez M.E., Pennell R.I., Meijer P.J., Ishikawa A., Dixon R.A., Lamb C. (1998). Reactive oxygen intermediates mediate a systemic signal network in the establishment of plant immunity. Cell.

[21] Martinez C., Baccou J.C., Bresson E., Baissac Y., Daniel J.F., Jalloul A., Montillet J.L., Geiger J.P., Assigbetsé K., Nicole M. (2000). Salicylic acid mediated by the oxidative burst is a key molecule in local and systemic responses of cotton challenged by an avirulent race of *Xanthomonas campestris* pv *malvacearum*. Plant Physiol..

[22] Tada Y., Spoel S.H., Pajerowska-Mukhtar K., Mou Z., Song J., Wang C., Zuo J., Dong X. (2008). Plant immunity requires conformational charges of NPR1 via S-nitrosylation and thioredoxins. Science.

[23] Blanco F., Salinas P., Cecchini N.M., Jordana X., van Hummelen P., Alvarez M.E., Holuigue L. (2009). Early genomic responses to salicylic acid in *Arabidopsis*. Plant Mol. Biol..

[24] Park S.W., Kaimoyo E., Kumar D., Mosher S., Klessig D.F. (2007). Methyl salicylate is a critical mobile signal for plant systemic acquired resistance. Science.

[25] Vernooij B., Friedrich L., Morse A., Reist R., Kolditz-Jawhar R., Ward E., Uknes S., Kessmann H., Ryals J. (1994). Salicylic acid is not the translocated signal responsible for inducing systemic acquired resistance but is required in signal transduction. Plant Cell.

[26] Attaran E., Zeier T.E., Griebel T., Zeier J. (2009). Methyl salicylate production and jasmonate signaling are not essential for systemic acquired resistance in *Arabidopsis*. Plant Cell.

[27] Maldonado A.M., Doerner P., Dixon R.A., Lamb C.J., Cameron R.K. (2002). A putative lipid transfer protein involved in systemic resistance signaling in *Arabidopsis*. Nature.

[28] Jung H.W., Tschaplinski T.J., Wang L., Glazebrook J., Greenberg J.T. (2009). Priming in systemic plant immunity. Science.

[29] Takashima Y., Ishiguri F., Iizuka K., Yoshizawa N., Yokota S. (2013). Proteome analysis of infection-specific proteins from Japanese birch (*Betula platyphylla* var. *japonica*) plantlet No.8 infected with Inonotus obliquus strain IO-U1. Plant Biotechnol..

[30] Takashima Y., Suzuki M., Ishiguri F., Iizuka K., Yoshizawa N., Yokota S. (2013). Cationic peroxidase related to basal resistance of *Betula platyphylla* var. * japonica* plantlet No.8 against canker-rot fungus *Inonotus obliquus* strain IO-U1. Plant Biotechnol..

[31] Rahman M.M., Ishiguri F., Takashima Y., Azad M.A.K., Iizuka K., Yoshizawa N., Yokota S. (2008). Anatomical and histochemical characteristics of Japanese birch (Tohoku) plantlets infected with the *Inonotus obliquus* IO-U1 strain. Plant Biotechnol..

[32] Murashige T., Skoog F. (1962). A revised medium for rapid growth and bio assays with tobacco tissue cultures. Physiol. Plant..

[33] Bradford M.M. (1976). A rapid and sensitive method for the quantitation of microgram quantities of protein utilizing the principle of protein-dye binding. Anal. Biochem..

[34] MS/MS Ions Search. http:www.matrixscience.com.

[35] Siedow J.N., Day D.A., Buchanan B.B., Gruissem W.G., Jones R.L. (2000). Respiration and photorespiration. Biochemistry & Molecular Biology of Plants.

[36] Segarra G., Casanova E., Bellido D., Odena M.A., Oliveira E., Trillas I. (2007). Proteome, salicylic acid, and jasmonic acid changes in cucumber plants inoculated with *Trichoderma asperellum* strain T34. Proteomics.

[37] Islam M.A., Sturrock R.N., Ekramoddoullah A.K.M. (2008). A proteomics approach to identify proteins differentially expressed in Douglas-fir seedlings infected by *Phellinus sulphurascens*. J. Proteomics.

[38] Tomaz T., Bagard M., Pracharoenwattana I., Lindén P., Lee C.P., Carroll A.J., Ströher E., Smith S.M., Gardeström P., Millar A.H. (2010). Mitochondrial malate dehydrogenase lowers leaf respiration and alters photorespiration and plant growth in *Arabidopsis*. Plant Physiol..

[39] Yao Y.-X., Dong Q.-L., Zhai H., You C.-X., Hao Y.-J. (2011). The functions of an apple *cytosolic malate dehydrogenase* gene in growth and tolerance to cold and salt stresses. Plant Physiol. Biochem..

[40] Siedow J.N., Day D.A., Buchanan B.B., Gruissem W.G., Jones R.L. (2000). Respiration and photorespiration. Biochemistry & Molecular Biology of Plants.

[41] Sako N., Stahmann M.A. (1972). Multiple molecular forms of enzymes in barley leaves infected with *Erysiphe graminis* f. sp. *hordei*. Physiol. Plant Pathol..

[42] Dennis D.T., Blakeley S.D., Buchanan B.B., Gruissem W.G., Jones R.L. (2000). Carbohydrate metabolism. Biochemistry & Molecular Biology of Plants.

[43] Azevedo R.A., Lea P.J. (2001). Lysine metabolism in higher plants. Amino Acids.

[44] Jenkinson C.P., Grody W.W., Cederbaum S.D. (1996). Comparative properties of arginases. Comp. Biochem. Physiol..

[45] Chen H., McCaig B.C., Melotto M., He S.Y., Howe G.A. (2004). Regulation of plant arginase by wounding, jasmonate, and the phtotoxin coronatine. J. Biol. Chem..

[46] Jones A.M.E., Thomas V., Bennett M.H., Mansfield J., Grant M. (2006). Modifications to the *Arabidopsis* defense proteome occur prior to significant transcriptional change in response to inoculation with *Pseudomonas syringae*. Plant Physiol..

[47] Kusano T., Yamaguchi K., Berberich T., Takahashi Y. (2007). Advances in polyamine research in 2007. J. Plant Res..

[48] Walters D.R. (2003). Polyamines and plant disease. Phytochemistry.

[49] Marina M., Maiale S.J., Rossi F.R., Romero M.F., Rivas E.I., Gárriz A., Ruiz O.A., Pieckenstain F.L. (2008). Apoplastic polyamine oxidation plays different roles in local responses of tabacco to infection by necrotrophic fungus *Sclerotinia sclerotiorum* and the biotrophic bacterium *Pseudomonas viridiflava*. Plant Physiol..

[50] Yoda H., Fujimura K., Takahashi H., Munemura I., Uchimiya H., Sano H. (2009). Polyamines as a common source of hydrogen peroxide in host- and nonhost hypersensitive response during pathogen infection. Plant Mol. Biol..

[51] Németh M., Janda T., Horváth E., Páldi E., Szalai G. (2002). Exogenous salicylic acid increases polyamine content but may decrease drought tolerance in maize. Plant Sci..

[52] Tzin V., Galili G. (2010). New insights into the shikimate and aromatic amino acids biosynthesis pathways in plants. Mol. Plant.

[53] Eberhard J., Ehrler T.T., Epple P., Felix G., Raesecke H.R., Amrhein N., Schmid J. (1996). Cytosolic and plastidic chorismate mutase isozymes from *Arabidopsis thaliana*: Molecular characterization and enzymatic properties. Plant J..

[54] Dixon R.A. (2001). Natural products and plant disease resistance. Nature.

[55] Pallas J.A., Paiva N.L., Lamb C., Dixon R.A. (1996). Tobacco plants epigenetically suppressed in phenylalanine ammonia-lyase expression do not develop systemic acquired resistance in response to infection by tobacco mosaic virus. Plant J..

[56] Shirasu K., Nakajima H., Rajasekhar V.K., Dixon R.A., Lamb C. (1997). Salicylic acid potentiates an agonist-dependent gain control that amplifies pathogen signals in the activation of defense mechanisms. Plant Cell.

[57] Smith-Becker J., Marois E., Huguet E.J., Midland S.L., Sims J.J., Keen N.T. (1998). Accumulation of salicylic acid and 4-hydroxybenzoic acid in phloem fluids of cucumber during systemic acquired resistance is preceded by a transient increase in phenylalanine ammonia-lyase activity in petioles and stems. Plant Physiol..

[58] Bächinger H.P. (1987). The influence of peptidyl-prolyl *cis-trans* isomerase on the *in vitro* folding of type III collagen. J. Biol. Chem..

[59] Miernyk J.A. (1999). Protein folding in the plant cell. Plant Physiol..

[60] Marvet J., Margis-Pinheiro M., Frendo P., Burkard G. (1994). Bean cyclophilin gene expression during plant development and stress conditions. Plant Mol. Biol..

[61] Walling L.L. (2006). Recycling or regulation? The role of amino-terminal modifying enzymes. Curr. Opin. Plant Biol..

[62] Cheng F.Y., Blackburn K., Lin Y.M., Goshe M.B., Williamson J.D. (2009). Absolute protein quantification by LC/MSn for global analysis of salicylic acid-induced plant protein secretion responses. J. Proteome Res..

[63] Delaney T.P., Uknes S., Vernooij B., Friendrich L., Weymann K., Negrotto D., Gaffney T., Gut-Rella M., Kessmann H., Ward E. (1994). A central role of salicylic acid in plant disease resistance. Science.

[64] Solomon M., Belenghi B., Delledonne M., Menachem E., Levine A. (1999). The involvement of cysteine proteases and protease inhibitor genes in the regulation of programmed cell death in plants. Plant Cell.

